# Unraveling the Dynamic Evolution of Volatile Aroma Compounds in Sea Buckthorn–Grape Composite Fruit Wine During Sequential Yeast–Lactic Acid Bacteria Fermentation

**DOI:** 10.3390/foods15132297

**Published:** 2026-06-26

**Authors:** Haixia Han, Chunkai Yu, Miao Zhang, Zhen Wang, Xiuli Yang, Jingjing Sun, Yue Cui, Zuoshan Feng, Mingxi Jia

**Affiliations:** 1 College of Food Science and Pharmacy, Xinjiang Agricultural University, Urumqi 830052, China; hanhaixia@xjau.edu.cn (H.H.); yck1318729228@163.com (C.Y.);; 2 Xinjiang Key Laboratory of Postharvest Fruit Science and Technology, Urumqi 830052, China; 3 College of Modern Industry in Chinese Herbal Medicine, Xinjiang Agricultural University, Urumqi 830052, China

**Keywords:** sea buckthorn, grapes, composite fruit wine, fermentation process, aroma components

## Abstract

Sea buckthorn is rich in functional components but features high acidity and susceptibility to oxidative deterioration, leading to flavor defects such as sour–astringent taste and rancidity in single-fruit wine. Co-fermentation is an effective strategy for flavor and nutrition complementarity, but the dynamic evolution of volatile aroma during yeast–lactic acid bacteria combined fermentation of such wine remains unclear. This study employed ‘Zhuangyuan Huang’ sea buckthorn and ‘Marselan’ grape to produce the composite fruit wine via sequential co-fermentation, and systematically investigated dynamic aroma changes using HS-SPME-GC-MS, orthogonal partial least squares discriminant analysis (OPLS-DA), and relative odor activity value (ROAV) analysis. Results revealed that post-fermentation, total detected volatile compounds increased from 90 to 118, with esters and alcohols rising by 24 and 11 respectively, serving as core contributors to enhanced aroma richness. ROAV analysis demonstrated this process significantly reduced the contribution of off-flavor acids, boosted the importance of floral, fruity, and sweet compounds, and elevated the sensory score from 26.8 to 84.1. OPLS-DA further confirmed significant inter-stage aroma differences with excellent intergroup discrimination. These findings confirm that this sequential fermentation breaks processing bottlenecks of high-acid fruits, reveals the synergistic flavor-modulating effects of multi-microbial sequential fermentation, and provides theoretical support for process optimization and high-value processing of composite fruit wine.

## 1. Introduction

Against the backdrop of the escalating global shift toward health-focused consumption, low-alcohol, nutrient-rich, and uniquely flavored fruit wines have gradually emerged as a new growth driver in the alcoholic beverage market. These products not only address consumers’ demand for healthier beverage options, but also offer a critical pathway for the value-added deep processing of specialty fruit and forestry resources [1,2]. In recent years, the fruit wine industry has experienced sustained expansion. Among its various segments, specialty composite fruit wines have emerged as a core direction for industrial upgrading, owing to their advantages of nutritional complementarity and a more complex flavor profile. Nevertheless, the industry currently confronts several critical bottlenecks, including severe product homogenization, insufficient deep-processing technologies, and low utilization efficiency of high-value raw materials. Against this context, unlocking the potential of local specialty raw materials through technological innovation, and developing differentiated products with core competitiveness, has become a pivotal issue for driving the high-quality development of the industry [3].

Sea buckthorn, a dual-purpose medicinal and edible plant, harbors a diverse array of functional components in its fruits, including vitamin C, polyphenols, and unsaturated fatty acids. It also exhibits prominent bioactivities such as antioxidant, anti-inflammatory, and immunomodulatory effects, demonstrating substantial commercial development value across the food, pharmaceutical, and nutraceutical industries [4,5]. Winemaking using sea buckthorn as the raw material represents a key development direction for the deep processing of this crop. During this process, the core nutritional components of the fruit can be well preserved, while microbial fermentation converts macromolecular substances into small-molecule active compounds with higher bioavailability for human absorption. Nevertheless, the inherent physicochemical properties of sea buckthorn fruits have severely hampered the market penetration of its wine products. Specifically, their organic acid content is far higher than that of conventional winemaking fruits, leading to a pronounced sour and astringent taste in single-ingredient sea buckthorn wine. Meanwhile, the abundant unsaturated fatty acids in the fruit are highly susceptible to oxidative rancidity, which generates an unpleasant rancid off-flavor. This issue severely impairs the sensory quality and market acceptability of the final products, and has become a core technical bottleneck hindering the large-scale development of the sea buckthorn wine industry [4,6]. While sea buckthorn wine has garnered a certain level of market demand, its inherent flavor defects have left its market share far below that of traditional fruit wines such as grape and apple wine. As such, there is an urgent need to break through this dilemma via targeted process innovation.

To tackle the flavor bottlenecks plaguing sea buckthorn wine, composite fermentation technology has emerged as a prominent research focus. Co-fermenting sea buckthorn with other flavor-complementary fruits enables synergistic improvements in acid neutralization, nutritional complementarity, and aroma layering [7]. As the most extensively and maturely utilized winemaking raw material globally, grape boasts a high sugar content that can effectively adjust the sugar–acid ratio of the fermentation system, and is rich in aroma precursors such as terpenes and esters. When co-fermented with sea buckthorn, this property successfully neutralized the latter’s high acidity, while enriching the aroma dimensions of the resulting fruit wine. This interaction delivers the well-known “1 + 1 > 2” synergistic quality enhancement effect. To date, notable progress has been achieved in research on various composite fruit wines, such as mulberry–grape and grape–blueberry variants. These studies have confirmed that composite fermentation can effectively ameliorate the flavor defects of single-fruit wines and enhance the sensory quality of the final products [8,9]. Nevertheless, research focusing on sea buckthorn–grape composite wine remains relatively scarce. Existing studies mostly center on the optimization of fermentation process parameters and static analysis of finished product quality, lacking a systematic elucidation of the dynamic evolution patterns of volatile aroma components across the entire fermentation process. Recent studies have demonstrated that mixed fermentation of complementary raw materials or multi-microbial sequential fermentation can effectively improve flavor complexity and nutritional quality of fruit wines. For example, Yu et al. [10] developed a novel Cyperus esculentus–raspberry composite wine and found that raw material complementation significantly increased esters and terpenes content, while Liu et al. [11] confirmed that non-Saccharomyces–Saccharomyces mixed fermentation could enhance aroma richness of cider by regulating microbial metabolic interactions. Aroma acts as the core sensory indicator of fruit wine quality, directly dictating consumer acceptability and the market competitiveness of the product [12]. Meanwhile, sequential co-fermentation technology employing yeast and lactic acid bacteria (LAB) has also been validated in multiple composite fruit wines to achieve precise flavor regulation through synergistic microbial metabolism [13]. Specifically, beyond the well-characterized malolactic fermentation that converts harsh malic acid to milder lactic acid to optimize taste balance, LAB exerts multifaceted biochemical functions in aroma formation: it can metabolize residual sugars and amino acids to produce secondary metabolites such as esters, higher alcohols, and volatile fatty acids, which directly enrich the fruity and floral aroma profile; it can also hydrolyze glycosidically bound aroma precursors to release free volatile compounds, thus enhancing aroma richness, while modulating off-flavor precursors to refine the overall sensory quality. However, within the sea buckthorn–grape composite wine system, the dynamic change characteristics of aroma substances during co-fermentation, as well as the synergistic regulation effects of the two microorganisms on aroma formation, have not been clearly elucidated to date. This research gap has severely constrained the process optimization and quality upgrading of such products, making it difficult to provide precise theoretical support for flavor optimization from the perspective of process regulation.

Against this backdrop, the present study employed sea buckthorn and grape as raw materials, and adopted sequential co-fermentation of yeast and LAB to brew the composite fruit wine, aiming to ameliorate the flavor defects of sea buckthorn wine through process innovation. Specifically, we employed Headspace Solid Phase Microextraction–Gas Chromatography–Mass Spectrometry (HS-SPME-GC-MS) to systematically elucidate the dynamic evolution patterns of volatile aroma components across the entire fermentation process. Combined with multivariate statistical analysis and ROAV analysis, we sought to identify the characteristic aroma differences and key flavor-contributing substances at different fermentation stages, and clarify the regulatory effects of co-fermentation on aroma formation. Findings from this work will not only provide a solid theoretical basis for the flavor regulation and new product development of sea buckthorn–grape composite wine, but also offer technical references for the value-added deep processing of specialty fruit and forestry resources. This work also holds important theoretical and practical significance for driving the high-quality development of related industries.

## 2. Materials and Methods

### 2.1. Materials and Reagents

Sea buckthorn (cultivar Zhuangyuan Huang, with total acid of 19.27 g/kg and total sugar of 5.23 g/100 g) was purchased from the planting base in Aheqi County, Kezhou Prefecture, Xinjiang, China; grape (cultivar Marselan, with total acid of 2.48 g/kg and total sugar of 21.85 g/100 g) was purchased from the planting base in Wujiaqu City, Xinjiang, China. Lalvin 71B yeast was purchased from Lallemand Biotech (Beijing, China) Co., Ltd. (Montreal, QC, Canada). Lactic acid bacteria (LAB), Oenococcus oeni, were purchased from Deboshi Home Brewing Machine Co., Ltd. (Yantai, Shandong, China). Sodium chloride and acetone were purchased from Xilong Chemical Co., Ltd. (Chengdu, Sichuan, China).

### 2.2. Raw Material Pretreatment

Preparation of sea buckthorn juice: Sound, uniformly mature sea buckthorn fruits were selected, with stems removed, before being crushed and pulped. The crude pulp was then diluted and mixed with pure water at a volume ratio of 1:1, and filtered through gauze to remove residues, yielding the raw sea buckthorn juice for later use.

Preparation of grape juice: Moldy and immature fruits were discarded, and the remaining grapes were de-stemmed and crushed, before being pressed using a mechanical press to extract the juice. The juice was filtered through four layers of gauze to remove peel residues, producing the raw grape juice for subsequent use.

The above-prepared sea buckthorn juice and grape juice were mixed at a volume ratio of 6:4 to obtain the composite juice. This ratio was selected based on our previous optimization study on sea buckthorn–grape composite wine fermentation, in which orthogonal tests using sensory score, acidity reduction rate, and alcohol content as evaluation criteria identified the 6:4 ratio as optimal [14]. White granulated sugar was then added to adjust the initial sugar content to 28° Bx, and food-grade calcium carbonate was supplemented to adjust the initial pH to 3.50, finally yielding the composite juice fermentation broth for subsequent fermentation experiments.

### 2.3. Sequential Co-Fermentation of Yeast and LAB

The present study employed the sequential co-fermentation process of yeast and LAB, with the specific procedure detailed below:

Alcoholic fermentation stage: In a preliminary experiment we conducted earlier to screen for the optimal wine yeast for a sea buckthorn–grape mixed system, Lalvin 71B demonstrated superior fermentation performance—higher alcohol conversion and stronger acid-reducing capacity—effectively reducing tartness and proving more suitable for sea buckthorn fruit wine [14]. The dry yeast powder of Lalvin 71B was weighed at an inoculation amount of 0.1%, and added into warm water at 37 °C with a sugar content of 2~3%, followed by static activation for 20 min. The activation was completed until uniform foam appeared on the liquid surface. The activated yeast solution was then fully inoculated into the composite juice fermentation broth, and alcoholic fermentation was carried out at a constant temperature of 20 °C for a fermentation period of 5 d.

Malolactic fermentation stage: LAB activation was performed 2 h before the end of alcoholic fermentation: The freeze-dried LAB powder was mixed with chlorine-free water at 20 °C at a mass–volume ratio of 1:10, and static placement for 15 min completed the activation. The activated LAB solution was then inoculated into the wine system after alcoholic fermentation at an addition amount of 0.05%. Malolactic fermentation was performed at a constant temperature of 20 °C for a period of 6 d, thus completing the entire co-fermentation process.

### 2.4. Wine Clarification Process

After the completion of fermentation, a bentonite–chitosan composite clarification process was employed to treat the wine. Specifically, at 21 °C, 1% (*w*/*v*) bentonite solution was added to the wine at a volume ratio of 0.05%, and 1% chitosan solution was supplemented at a volume ratio of 0.03% simultaneously. After mixing, the mixture was left to stand and clarify for 30 h, followed by filtration to remove the precipitate, finally yielding the clarified finished sea buckthorn–grape composite fruit wine.

### 2.5. Sensory Evaluation

Sensory evaluation was performed based on the method described by Lan et al. [15] with modifications. A sensory panel consisting of 10 experts (5 females and 5 males, aged 25 to 40 years, from the College of Viticulture and Enology, Xinjiang Agricultural University) was recruited to evaluate the composite fruit wine samples. All panelists had more than 3 years of experience in sensory evaluation of various beverages, including Baijiu and wine. Prior to the sensory evaluation, these 10 panelists underwent a 2-month training program with 4 h per week, using the “Le Nez du Vin” aroma kit (54 aromas; Yixiangle, Hong Kong, China) [16]. The evaluation was conducted in a well-ventilated, odor-free, and quiet room, and distilled water mouthwash was provided for the panelists between consecutive sample evaluations to eliminate sensory carryover effects. The sample presentation order was fully randomized across all panelists and evaluation sessions to prevent potential order biases. The panelists evaluated the composite fruit wine samples at different fermentation stages in terms of 5 sensory attributes, including appearance, color, aroma, taste, and typicality. Each panelist scored the samples independently in the aforementioned order, and the average score was calculated. All samples adopted in our sensory analysis are common commercially available edible fruits and fruit wine products without special functional additives or unconventional raw materials; the tasting process only involves routine oral ingestion of regular food under normal dining scenarios, bringing no physical discomfort, health risks or privacy collection to participating panelists. The whole sensory trial belongs to conventional food quality sensory characterization without invasive human-related detection or special human intervention designs, which is the core reason why our research qualifies for ethical exemption. The sensory evaluation criteria are presented in Table 1.

### 2.6. Determination of Volatile Aroma Components by HS-SPME-GC-MS

In this study, the Headspace Solid Phase Microextraction–Gas Chromatography–Mass Spectrometry (HS-SPME-GC-MS, Agilent, Santa Clara, CA, USA) was used for the determination of volatile aroma components. The device was equipped with a 65 μm PDMS/DVB extraction fiber (Guangzhou Zhida Co., Ltd., Guangzhou, Guangdong, China). The detection procedure was as follows: 3 mL of liquid sample was added into a 20 mL headspace vial, followed by incubation at 70 °C for 40 min with an agitation speed of 500 rpm. Then SPME extraction was performed, with an extraction depth of 35 mm, an extraction time of 40 min, and a desorption time of 500 s.

A DB-5MS chromatographic column was adopted, with specifications of 30 m × 0.25 mm × 0.25 μm. The Gas Chromatography (GC) conditions were set as follows: inlet temperature of 250 °C; temperature program: initial temperature of 40 °C, held for 1 min, then increased to 310 °C at a rate of 5 °C/min, and held for 5 min; carrier gas: helium (99.999%), flow rate: 1.0 mL/min, splitless injection. The Mass Spectrometry (MS) conditions were set as follows: EI ion source with electron energy of 70 eV; transmission line temperature of 250 °C; ion source temperature of 230 °C, quadrupole temperature of 150 °C; mass scan range of 50~600 u; scan mode: full scan; solvent delay of 1 min.

### 2.7. Qualitative and Quantitative Analysis of Volatile Substances

The mass spectrometry data were processed using the Xcalibur workstation. Qualitative analysis of volatile compounds was comprehensively confirmed via dual validation strategies: matching with the National Institute of Standards and Technology 14 (NIST14, Gaithersburg, MD, USA) standard mass spectral library (only compounds with a similarity match score > 80% were reserved) and cross-verification with reference retention indices (RIs) reported in the published peer-reviewed literature, which guaranteed the accuracy and reliability of compound identification. Notably, the quantitative analysis in this study was performed using peak area normalization to obtain the relative percentage content of each volatile compound, rather than internal standards or calibration curves for absolute quantification. This approach was adopted because the primary focus of this work was to compare the relative dynamic changes of volatile compounds across different fermentation stages, and all samples were processed and detected under identical experimental conditions, ensuring the reliability of relative comparisons between samples and between compounds.

### 2.8. Analysis of Relative Odor Activity Value (ROAV)

To clarify the contribution of each volatile substance to the overall flavor of the sample, the ROAV evaluation was adopted in this study [17]. Notably, all volatile compounds included in the ROAV calculation have published sensory odor thresholds. The sensory thresholds and aroma descriptions of each volatile substance were obtained by reviewing the published literature [18,19,20,21]. The calculation formula of ROAV is as follows:

(1) $$\mathrm{ROAV}=100\times \frac{C_{i}}{\mathrm{Cmax}}\times \frac{\mathrm{Tmax}}{T_{i}}$$

where C_i_ is the relative content of the i-th volatile substance (%); T_i_ is the sensory threshold of the i-th volatile substance (μg/L); Cmax is the relative content of the substance with the largest contribution to the overall flavor of the sample (%); Tmax is the sensory threshold of the substance with the largest contribution to the overall flavor of the sample (μg/L). According to the ROAVs, the contribution of flavor substances was classified as follows: substances with ROAV ≥ 1 were the key flavor substances of the sample, which played a major contribution role to the overall flavor; substances with 0.1 ≤ ROAV < 1 were important modifying flavor substances, which played an important modifying role in the flavor richness of the sample; the larger the ROAV, the stronger the contribution of the substance to the overall flavor, and the calculation results of all substances satisfied 0 ≤ ROAV ≤ 100.

### 2.9. Statistical Analysis

All experiments in this study were set with 3 parallel replicates, and the experimental results were expressed as mean ± standard deviation (SD). One-way ANOVA was performed using SPSS 26.0 software, and Duncan’s multiple range test was used for difference significance test, with *p* < 0.05 as the criterion for significant difference. The differential circular heatmap was drawn using the Chiplot online plotting platform (https://www.chiplot.online); Orthogonal Partial Least Squares Discriminant Analysis (OPLS-DA) was performed using SIMCA 14.1 software. Prior to model construction, all volatile compounds were subjected to unit variance scaling. Seven-fold cross-validation and 200-times permutation testing were conducted to validate the robustness of the model and exclude potential overfitting, aiming to clarify the differences in aroma characteristics of samples at different fermentation stages.

## 3. Results

### 3.1. Dynamic Evolution Characteristics of Major Volatile Aroma Substances During Co-Fermentation

During the sequential co-fermentation of yeast and LAB for sea buckthorn–grape composite fruit wine, the content and composition of different categories of volatile aroma substances showed differentiated dynamic evolution patterns, which jointly drove the construction and optimization of the flavor system of the fruit wine [22].

**Figure 1:**
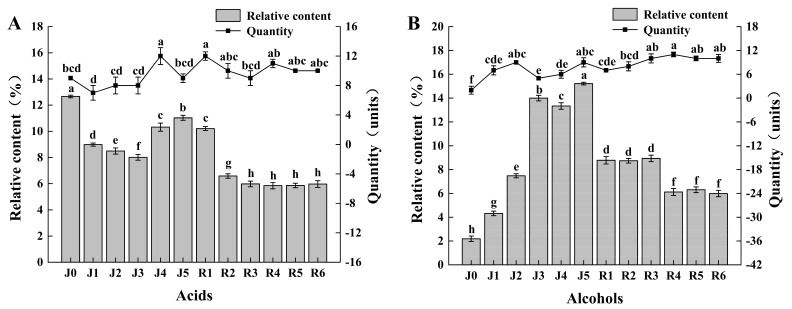
Analysis of changes in acidic and alcoholic aroma substances during fruit wine fermentation. (**A**) Acidic substances; (**B**) alcoholic substances. J0 represents sea buckthorn–grape blended juice; J1–J5 represent the yeast fermentation stage; R1–R6 represent the LAB fermentation stage. *n* = 3 per group. Data were presented as mean ± SD. Different letters above bars indicate statistically significant differences (*p* < 0.05) by one-way ANOVA followed by Duncan’s multiple range test.

The content and composition of acidic substances are the core factors regulating the sensory acidity and flavor balance of fruit wine, and their dynamic changes directly determine the taste coordination of the product [1]. As shown in Figure 1A, the relative content of acidic substances first decreased and then increased during the yeast fermentation stage, and continued to decrease during the LAB fermentation stage, which was consistent with the acid-reducing process of LAB metabolizing malic acid into lactic acid. The number of detected acidic substances showed slight fluctuations: 9 types were detected in the initial sea buckthorn–grape composite juice; the number remained 9 at the end of yeast fermentation, and increased to 10 at the end of LAB fermentation.

Alcohols are key aroma-active components of fruit wine. They can not only directly endow the product with floral and fruity aromas, but also act as precursor substances to participate in ester synthesis, which are crucial for the construction of the overall aroma system [10,17]. As shown in Figure 1B, the relative content of alcohols increased continuously during the yeast fermentation stage, mainly attributed to the glycolysis and amino acid deamination and decarboxylation metabolism of yeast. During the LAB fermentation stage, the relative content gradually decreased, and it was speculated that part of the alcohols was converted into esters or other secondary metabolites during this process. The detection number of alcohols increased gradually: only 2 types were detected in the initial composite juice; the number increased to 8 at the end of yeast fermentation, and further rose to 10 after LAB fermentation, indicating that co-fermentation significantly enriched the types of alcohols, providing a more abundant flavor foundation for fruit wine.

**Figure 2:**
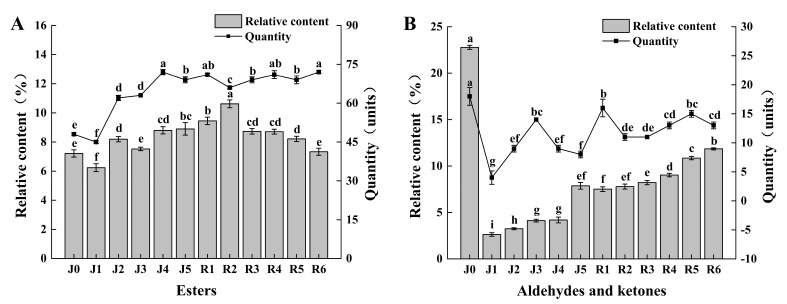
Analysis of changes in ester and aldehyde/ketone aroma substances during fruit wine fermentation. (**A**) Esters substances; (**B**) aldehydes and ketones substances. J0 represents sea buckthorn–grape blended juice; J1–J5 represent the yeast fermentation stage; R1–R6 represent the LAB fermentation stage. *n* = 3 per group. Data were presented as mean ± SD. Different letters above bars indicate statistically significant differences (*p* < 0.05) by one-way ANOVA followed by Duncan’s multiple range test.

Esters are the core components of fruit wine aroma, most of which carry fresh floral and fruity notes, playing a decisive role in the formation of the overall flavor [23]. As shown in Figure 2A, the relative content of esters increased continuously during the yeast fermentation stage, mainly driven by the alcohol–acid esterification reaction during yeast metabolism. During the LAB fermentation stage, it showed a fluctuation of first increasing and then decreasing, indicating that LAB metabolism also participated in the synthesis and transformation of esters. The detection number of esters increased continuously: 48 types were detected in the initial composite juice; the number increased to 69 at the end of yeast fermentation, and further rose to 72 at the end of LAB fermentation, indicating that co-fermentation greatly enriched the composition of esters, which was the core reason for the improvement of fruit wine’s aroma richness.

Aldehydes and ketones have low absolute content in fruit wine, but they generally have low odor thresholds, thus playing an important modifying role in the overall aroma [24]. As shown in Figure 2B, the relative content of aldehydes and ketones in the initial composite juice was relatively high, reaching 22.78%. During the yeast fermentation stage, their content first decreased sharply and then rebounded steadily, which might be attributed to the reduction of aldehydes by yeast at the initial fermentation stage, as well as the generation of new aldehydes and ketones via amino acid metabolism at the later stage. During the LAB fermentation stage, the relative content continued to increase. The detection number of aldehydes and ketones first decreased and then fluctuated rising: 18 types were detected in the initial composite juice; the number decreased to 8 at the end of yeast fermentation, and rebounded to 13 at the end of LAB fermentation, indicating that part of the aldehydes and ketones inherent in raw materials were consumed by microorganisms during fermentation, and microorganisms may have synthesized new such substances through metabolism.

**Figure 3:**
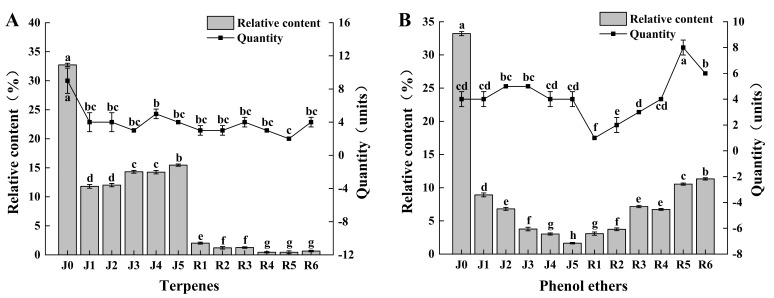
Analysis of changes in terpenes and phenolic substances during fruit wine fermentation. (**A**) Terpenes substances; (**B**) phenolic substances. J0 represents sea buckthorn–grape blended juice; J1–J5 represent the yeast fermentation stage; R1–R6 represent the LAB fermentation stage. *n* = 3 per group. Data were presented as mean ± SD. Different letters above bars indicate statistically significant differences (*p* < 0.05) by one-way ANOVA followed by Duncan’s multiple range test.

Terpenes include monoterpenes and sesquiterpenes, among which monoterpenes can endow fruit wine with unique floral and citrus-like aromas, serving as important characteristic aroma substances [25]. As shown in Figure 3A, the relative content of terpenes in the initial composite juice was relatively high, reaching 32.73%. During the yeast fermentation stage, their content first decreased sharply and then rebounded steadily, which might be attributed to the hydrolysis of partial terpene glycosides and the metabolic consumption of free terpenes at the initial fermentation stage. During the LAB fermentation stage, the relative content continued to decrease. The detection number of terpenes first decreased and then fluctuated stably: 9 types were detected in the initial composite juice; the number decreased to 4 at the end of yeast fermentation, and remained 4 at the end of LAB fermentation, indicating that part of the terpenes inherent in raw materials were consumed during fermentation, and the remaining 4 types became the characteristic terpene aroma components of the fruit wine.

Although phenols and ethers account for a low proportion in fruit wine aroma, they contribute significantly to the richness and uniqueness of flavor [26,27]. Among them, some phenols can endow the product with spicy, smoky and nutty notes, while most ethers have fresh floral and fruity aromas, effectively improving the flavor hierarchy. As shown in Figure 3B, the relative content of phenols and ethers in the initial composite juice was relatively high, reaching 33.22%. The content decreased continuously during the yeast fermentation stage, and then rebounded continuously during the LAB fermentation stage. The detection number of phenols and ethers showed a fluctuation of first increasing, then decreasing and then rising again: 4 types were detected in the initial composite juice; the number remained 4 at the end of yeast fermentation, and increased to 6 at the end of LAB fermentation, indicating that LAB metabolism further enriched the composition of phenols and ethers, bringing more unique flavor characteristics to the fruit wine.

### 3.2. Analysis of the Differences in Composition and Content of Volatile Aroma Substances Among Different Fermentation Stages

As shown in Table S1, 9 acids, 2 alcohols, 48 esters, 9 terpenes, 18 aldehydes and ketones, and 4 phenols and ethers were identified in the sea buckthorn–grape composite juice. Compounds with a relative content exceeding 1% for acidic substances, they included valeric acid, caprylic acid, nonanoic acid, cis-5-dodecenoic acid; for esters, they included methyl propionate, ethyl isovalerate, methyl acetoacetate, ethyl hexanoate, isoamyl n-butyrate, 3-methylbutyl 2-methylbutanoate, 3-methylbutyl 3-methylbutanoate, ethyl benzoate, hexyl 2-methylbutyrate, ethyl phenylacetate, 2-methyl butyl hexanoate, ethyl trans-4-decenoate, ethyl decanoate, benzyl valerate, isopentyl octanoate, 2-phenylethyl-2-methylbutyrate; for terpenes, they included ocimene, 3-carene; for aldehydes and ketones, they included 3-furaldehyde, 3,4-dimethyl-benzaldehyde; and phenols and ethers: 1-(1,1-Dimethylethoxy)-2-methylpropane.

After yeast fermentation was complete, 9 acids, 8 alcohols, 69 esters, 4 terpenes, 8 aldehydes and ketones, and 4 phenols and ethers were identified in the fruit wine. The compounds with a relative content exceeding 1% were: acidic substances (caprylic acid, cis-5-dodecenoic acid, decanoic acid), alcohols (1-pentanol), esters (ethyl hexanoate, 3-methylbutyl 3-methylbutanoate, ethyl trans-4-decenoate, isopentyl octanoate, 2-phenylethyl-2-methylbutyrate, ethyl laurate, 2-phenethyl hexanoate, propyl lactate, isoamyl acetate, isoamyl caproate, phenethyl acetate, ethyl dec-9-enoate, ethyl 8-nonenoate, pentyl benzoate), terpenes (styrene) and aldehydes and ketones (2,3-pentanedione).

Following the completion of LAB fermentation, 10 acids, 10 alcohols, 72 esters, 4 terpenes, 13 aldehydes and ketones, and 6 phenols and ethers were identified in the fruit wine. Among these, the compounds with a relative content exceeding 1% include: acidic substances (cis-5-dodecenoic acid, decanoic acid), esters (ethyl hexanoate, 3-methylbutyl 3-methylbutanoate, ethyl decanoate, isopentyl octanoate, ethyl laurate, ethyl myristate, ethyl cis-9-hexadecenoate, ethyl palmitate, methyl formate, isoamyl acetate, hexyl acetoacetate, isoamyl caproate, pentyl benzoate, ethyl 9-hexadecenoate, diethyl succinate, monoethyl succinate, ethyl caprylate, isoamyl benzoate), aldehydes and ketones (3,4-dimethyl-benzaldehyde, 2,4-dimethylbenzaldehyde).

**Figure 4:**
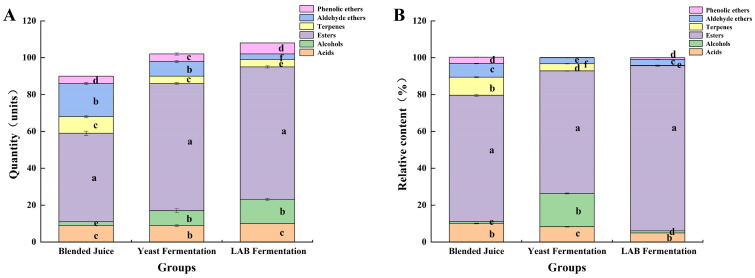
Stacked bar chart analysis of aroma compounds in sea buckthorn–grape blend wine. (**A**) Distribution of the number of aroma compounds; (**B**) relative content of aroma compounds. *n* = 3 per group. Data were presented as mean ± SD. Different letters above bars indicate statistically significant differences (*p* < 0.05) by one-way ANOVA followed by Duncan’s multiple range test.

The stacked column chart (Figure 4) showed that: in terms of quantity, the composite juice had more acidic substances, esters, terpenes and aldehydes and ketones; after yeast fermentation, there were more acidic substances, esters, alcohols and aldehydes and ketones; after LAB fermentation, there were more acidic substances, alcohols, esters and phenols and ethers. In terms of relative content, acidic substances, esters, terpenes and aldehydes and ketones accounted for a relatively high proportion in the composite juice; after yeast fermentation, acidic substances, alcohols and esters accounted for a relatively high proportion; after LAB fermentation, acidic substances, esters and aldehydes and ketones accounted for a relatively high proportion.

### 3.3. Analysis of Aroma Differential Substances During Fermentation Stages Based on Multivariate Statistics

**Figure 5:**
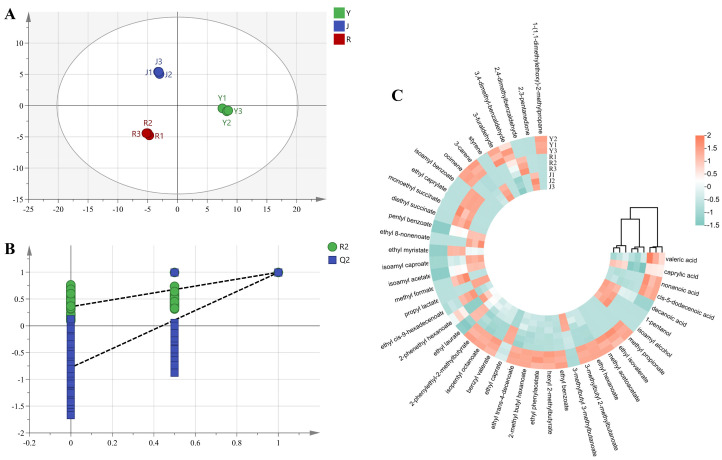
Multivariate statistical analysis and visualization of aroma compounds. (**A**) OPLS-DA analysis of aroma compounds; (**B**) model cross-validation results; (**C**) heatmap of aroma compounds with relative concentrations > 1%. Note: Y: blended fruit juice; J: yeast-fermented blended fruit wine; R: LAB-fermented blended fruit wine. *n* = 3 per group. Data were presented as mean ± SD.

This OPLS-DA analysis adopted all 118 detectable volatile aroma compounds as dependent variables after unit variance scaling to remove dimensional deviations, with three fermentation-related sample groups (composite juice, yeast-fermented fruit wine, LAB-fermented fruit wine) defined as independent variables (Figure 5A,B). The model achieved robust statistical performance (R^2^X = 0.954, R^2^Y = 0.998, Q^2^ = 0.996), and the Q^2^ value validated via 7-fold cross-validation guaranteed reliable predictive power. These high statistical indicators originated from distinct aroma metabolic variations across fermentation stages rather than model overfitting. Furthermore, 200 permutation tests were conducted for robustness verification; all permuted models presented much lower R^2^Y and Q^2^ values with a negative Q^2^ regression intercept, excluding overfitting and random correlation. The score plot also exhibited favorable intra-group aggregation and significant inter-group separation of all samples. The composite juice was located in the fourth quadrant, the yeast-fermented fruit wine was located in the second quadrant, and the LAB-fermented fruit wine was located in the third quadrant, indicating that co-fermentation could effectively change the volatile aroma component profile, and these differences could be used as important indicators to distinguish the quality of fruit wine. The differential heatmap (Figure 5C) was drawn with aroma substances with relative content greater than 1% and VIP value greater than 1, and the color depth reflected the content level (blank indicated not detected). The results showed that: methyl propionate, ethyl isovalerate, methyl acetoacetate, ethyl hexanoate, ethyl phenylacetate, isopentyl octanoate, nonanoic acid and other substances were dominant in the composite juice with significant differences; 1-pentanol, isoamyl alcohol, decanoic acid, isoamyl caproate, ethyl 8-nonenoate and other substances were dominant in the yeast-fermented fruit wine; isoamyl benzoate, ethyl caprylate, monoethyl succinate, diethyl succinate and other substances were dominant in the LAB-fermented fruit wine. Cluster analysis divided the samples into three categories, which further confirmed that there were significant differences in the aroma composition among the composite juice, yeast-fermented fruit wine and LAB-fermented fruit wine.

### 3.4. Evolution Analysis of Flavor Contribution of Key Aroma Substances Based on ROAV

Based on the ROAVs (Table 2), the key flavor substances at each stage were evaluated (ROAV ≥ 1 indicated major contributors, and 0.1 ≤ ROAV < 1 indicated modifying substances). In the composite juice, acidic substances valeric acid (ROAV = 1.38), caprylic acid (6.10), nonanoic acid (13.26) were the main flavor contributors, endowing the system with cheesy, soapy, oily and sweaty acid odors; (methylthio)acetic acid (0.41), isovaleric acid (0.76), heptanoic acid (0.45) played a modifying role, presenting sulfury, cheesy, sweaty acid and other odors. These acidic substances were important reasons for the unpleasant odor of sea buckthorn products. Among alcohols, 1-octanol (0.51) and phenylethyl alcohol (0.26) played a modifying role, providing oily, fruity and floral aromas. Among esters, ethyl isovalerate (13.66), ethyl hexanoate (100), hexyl 2-methylbutyrate (12.31) and other substances had ROAV > 10, contributing floral, fruity and oily aromas. Among terpenes, α-phellandrene (16.87), (E)-3,7-dimethylocta-1,3,6-triene (63.89), 2,6-dimethyl-2,4,6-octatriene (43.59), aristolene (72.69) and other substances had ROAV > 10, contributing floral, fruity and grassy aromas. Among aldehydes and ketones, heptaldehyde (80.37), benzaldehyde (33.65), (2E)-2-nonenal (53.82) and other substances had ROAV > 10, contributing floral, fruity and nutty aromas. Among phenols and ethers, 2,4-di-tert-butylphenol (1.48), ethyl propyl ether (2.81), 1-(1,1-dimethylethoxy)-2-methylpropane (2.26) were the main contributors, providing camphoraceous, herbal and fruity aromas.

After yeast fermentation, the aroma composition changed significantly. Among acidic substances, caprylic acid (2.65), decanoic acid (18.03), lauric acid (5.86) were the main contributors, providing oily, waxy and fruity aromas; the original unpleasant acidic substances disappeared or their ROAVs decreased significantly, indicating that fermentation effectively improved the off-flavor of sea buckthorn. Among alcohols, 1-pentanol (4.57), 2-(methylthio)ethanol (5.35) were the main contributors, providing alcoholic, sweet, oily, onion-like and other odors. Among esters, 3-methylbutyl 3-methylbutanoate (13.42), isoamyl caproate (66.10), ethyl dec-9-enoate (54.22) and other substances had ROAV > 10, contributing floral, fruity and oily aromas. Among terpenes, styrene (9.22), farnesol (1.03) were the main contributors, providing floral, fruity and woody aromas. Among aldehydes and ketones, benzaldehyde (5.72) was the main contributor, providing sweet and bitter almond odors. Among phenols and ethers, (E)-2-methoxy-4-(1-propenyl)phenol (3.19) was the main contributor, providing spicy, smoky and vanilla odors.

After LAB fermentation, the aroma characteristics were further optimized. Among acidic substances, decanoic acid (3.36) was the main contributor, providing oily, waxy and fruity aromas; the ROAVs of unpleasant flavor acids further decreased. Among alcohols, trans-nerolidol (1.25) was the main contributor, providing floral, fruity and woody aromas. Among esters, ethyl decanoate (41.37), ethyl laurate (14.64), ethyl cis-9-hexadecenoate (25.44) and other substances had ROAV > 10, contributing floral, fruity and oily aromas. The ROAVs of all terpenes were < 0.1, with a weak effect on the flavor. Among aldehydes and ketones, 3,4-dimethyl-benzaldehyde (9.30), 2H-pyran-2,6(3H)-dione (43.16), 2,4-dimethylbenzaldehyde (8.58) and other substances were the main contributors, providing sweet and bitter almond odors. Among phenols and ethers, 2,4-di-tert-butylphenol (1.20), (E)-2-methoxy-4-(1-propenyl)phenol (1.00) were the main contributors, providing herbal, smoky and vanilla odors. Overall, co-fermentation significantly reduced the contribution of off-flavor substances, and improved the relative importance of floral–fruity and sweet aroma substances.

**Table 2:** ROAVs of volatile flavor compounds in blended fruit juices, yeast-fermented fruit wines, and LAB-fermented fruit wines.

Category	Number	Name	CAS	Threshold	Smell	ROAV		
						Composite Juice	Yeast Fermentation	LAB Fermentation
Acids	1	(Methylthio)acetic acid	2444-37-3	2	Sulfurous, onion, putrid	0.41		
	2	Isovaleric acid	503-74-2	5	Cheesy, fruity, sweaty	0.76		
	3	Valeric acid	109-52-4	10	Cheesy, sweaty acidic	1.38	0.14	
	4	Heptanoic acid	111-14-8	5	Oily, cheesy, fruity	0.45		
	5	Octanoic aci	124-07-2	2	Waxy, oily	6.10	2.65	0.16
	6	Nonanoic acid	112-05-0	1	Soapy, oily, fruity	13.26	0.17	0.21
	7	cis-5-Dodecenoic acid	2430-94-6	10	Floral, fruity, oily	3.00	0.52	0.26
	8	Hexanoic acid	142-62-1	2	Cheesy, sweaty		0.8	0.44
	9	2-Methylhexanoic acid	4536-23-6	0.5	Fruity, cheesy, oily		0.4	
	10	Decanoic acid	334-48-5	0.5	Oily, waxy, fruity		18.03	3.36
	11	Lauric acid	143-07-7	0.1	Soapy, oily, waxy		5.86	
	12	2-Methylhexanoic acid	116-53-0	2	Apple, banana			0.15
	13	Ethyl (Z)-4-decenoate	7367-84-2	2	Peach, apricot, stone fruit			0.25
Alcohols	1	1-Octanol	111-87-5	5	Oily, waxy, fruity	0.51		
	2	Phenethyl alcohol	60-12-8	20	Rose, sweet	0.26		
	3	1-Pentanol	71-41-0	10	Alcoholic, sweet, oily		4.57	
	4	2-Methylbutanol	137-32-6	5	Fruity, alcoholic, spicy		0.31	
	5	Isoamyl alcohol	123-51-3	5	Alcoholic, banana, fruit		0.55	
	6	2-(Methylthio)ethanol	5271-38-5	0.1	Sulfurous, onion, sweet		5.35	
	7	3-(Methylthio)propanol	505-10-2	0.5	Corn, potato, sulfurous		0.73	0.37
	8	1-Heptanol	111-70-6	1	Oily, floral, alcoholic			0.12
	9	trans-Nerolidol	40716-66-3	0.1	Floral, fruity, woody			1.25
Esters	1	Methyl propionate	554-12-1	10	Fruity, sweet, ethereal	0.98		
	2	Ethyl isovalerate	108-64-5	2	Banana, apple, fermented	13.66		
	3	Methyl acetoacetate	13865-19-5	5	Sweet, fruity, caramel	2.97		
	4	Propyl isovalerate	557-00-6	3	Fruity, sweet, cheesy	0.61		
	5	Ethyl hexanoate	123-66-0	1	Apple, banana, oily	100	7.68	5.52
	6	Isobutyl isovalerate	589-59-3	2	Fruity, sweet, spicy	1.63		
	7	Isoamyl isobutyrate	2050-01-3	1	Fruity, sweet, fermented	7.05	0.16	
	8	Isoamyl butyrate	106-27-4	2	Banana, pineapple, sweet	4.09	0.12	
	9	2-Methylbutyl butyrate	51115-64-1	1	Fruity, sweet, green grass	6.12		
	10	cis-3-Hexenyl isovalerate	35154-45-1	0.5	Fruity, floral, green plant	6.10		
	11	3-Methylbutyl 2-methylbutanoate	27625-35-0	1	Fruity, sweet, spicy	14.93	4.49	
	12	Isoamyl isovalerate	659-70-1	2	Banana, apple, fermented	7.01	13.42	2.38
	13	Methyl octanoate	111-11-5	0.5	Fruity, oily, floral	10.07		
	14	Butyl hexanoate	626-82-4	1	Fruity, sweet, oily	2.76		
	15	Pentyl 3-methylbutanoate	25415-62-7	1	Fruity, sweet, spicy	3.00	1.13	
	16	Ethyl benzoate	93-89-0	1	Floral, sweet, honey	55.44	2.2	1.31
	17	Hexyl 2-methylbutanoate	10032-13-0	1	Fruity, sweet, oily	12.31	0.5	0.11
	18	Furfuryl valerate	36701-01-6	1	Roasted, nutty, fruity	4.95		1.98
	19	Furfuryl isovalerate	13678-60-9	0.5	Roasted, nutty, fruity	10.73	0.49	0.19
	20	Ethyl phenylacetate	101-97-3	0.5	Rose, sweet	43.75	1.78	1.49
	21	2-Methylbutyl hexanoate	2601-13-0	22	Fruity, oily	1.73	0.12	
	22	Propyl benzoate	2315-68-6	3	Floral, sweet, vanilla	0.51		0.32
	23	Pentyl hexanoate	540-07-8	1	Fruity, sweet, oily	1.18		
	24	Propyl octanoate	624-13-5	0.5	Fruity, oily, floral	1.90	0.35	
	25	Ethyl nonanoate	123-29-5	0.5	Fruity, oily, floral, waxy	4.49	4.37	1.9
	26	Isobutyl benzoate	120-50-3	3	Floral, sweet, spicy	0.17		
	27	2-Hydroxyethyl benzoate	94-33-7	0.5	Cherry, strawberry, floral	0.89		
	28	Isoamyl 2-furoate	615-12-3	1	Roasted, nutty, fruity	1.30		
	29	Methyl decanoate	110-42-9	0.5	Fruity, oily, floral, waxy	1.47	0.18	
	30	Heptyl isovalerate	56423-43-9	0.2	Apple, peach, floral	12.78	0.79	
	31	cis-3,7-Dimethyl-2,6-octadien-1-yl acetate	141-12-8	0.1	Fruity, floral, citrus	11.95		
	32	Butyl benzoate	136-60-7	2	Floral, sweet, vanilla	0.13		
	33	Ethyl trans-4-decenoate	76649-16-6	0.5	Fruity, oily, floral	51.36	6.44	1.08
	34	Hexyl hexanoate	6378-65-0	1	Fruity, sweet, oily	1.48		
	35	Ethyl decanoate	110-38-3	0.5	Fruity, oily, floral, waxy	67.47	5.03	41.37
	36	Benzyl valerate	10361-39-4	1	Fruity, sweet, spicy	22.14	2.1	0.85
	37	Isoamyl octanoate	2035-99-6	0.5	Fruity, oily, floral	43.93	10.44	4.04
	38	2-Methylbutyl octanoate	67121-39-5	0.1	Clove	47.22	17.04	5.86
	39	Ethyl 2,4-decadienoate	3025-30-7	0.1	Fruity, oily, floral, nutty	11.62	2.48	
	40	2-Phenylethyl 2-methylbutanoate	24817-51-4	0.5	Fruity, sweet, rose	43.13	7.47	2.5
	41	Pentyl salicylate	2050-08-0	1	Floral, sweet, herbal	1.51	0.19	
	42	Ethyl dodecanoate	106-33-2	1	Fruity, oily, floral, waxy	6.80	17.31	14.64
	43	2-Phenylethyl hexanoate	6290-37-5	0.5	Fruity, sweet, rose	13.19	6.08	
	44	Benzyl benzoate	120-51-4	2	Floral, sweet, spicy	0.24		
	45	Ethyl myristate	124-06-1	0.1	Apple, banana, floral	7.82	11.75	15.42
	46	2-Phenylethyl benzoate	94-47-3	0.5	Floral, sweet, rose	0.61		0.23
	47	Ethyl cis-9-hexadecenoate/Ethyl palmitoleate	56219-10-4	0.1	Fruity, oily, floral, waxy	21.41	7.54	25.44
	48	Ethyl palmitate	628-97-7	0.1	Orange, grapefruit, citrus	31.31	6.07	18.26
	49	Propyl lactate	616-09-1	50	Fruity, milky		0.16	
	50	Methyl isothiocyanate	556-61-6	1	Mustard, horseradish		0.12	
	51	Isoamyl acetate	123-92-2	20	Banana		0.95	0.32
	52	Methyl dithioacetate	2168-84-5	0.1	Sulfurous odor		2.21	
	53	Hexyl acetate	142-92-7	1	Apple, pear		0.17	
	54	Ethyl 2-furoate	614-99-3	0.2	Caramel, nutty		2.36	0.8
	55	Ethyl heptanoate	106-30-9	0.5	Peach, apricot		1.13	0.77
	56	Isoamyl valerate	2050-09-1	0.3	Strawberry, raspberry		1.77	0.49
	57	2-Methylbutyl isovalerate	2445-77-4	0.05	Apple, banana		1.12	
	58	Isobutyl hexanoate	105-79-3	0.3	Apple, peaches		0.71	
	59	Octyl 2-methylbutanoate	29811-50-5	0.08	Tropical fruit, oily		2.93	
	60	Citronellyl formate	105-85-1	0.3	Vanilla		0.22	0.49
	61	Hexyl acetoacetate	13562-84-0	0.1	Fruity, floral		4.08	
	62	Isoamyl hexanoate	2198-61-0	0.2	Fruity, floral		66.10	10.59
	63	2-Phenylethyl acetate	103-45-7	0.1	Rose		100	100
	64	Heptyl acetate	112-06-1	0.2	Apple, pear		0.7	
	65	Nonyl acetate	143-13-5	0.1	Citrus, floral		1.96	
	66	2-methylpropyl octanoate	5461-06-3	0.2	Citrus, floral		3.63	0.51
	67	Pentyl octanoate	638-25-5	0.2	Apple, peach, floral		3.98	0.78
	68	Geranyl isovalerate	109-20-6	0.01	Rose, lavender, citrus		8.78	
	69	Ethyl dec-9-enoate	67233-91-4	0.06	Orange, grapefruit, floral		54.22	23.5
	70	Methyl 2-oxobutanoate	3952-66-7	0.2	Apple, pear, oily		4.26	
	71	2-Phenylethyl isovalerate	140-26-1	0.02	Rose		16.27	59.53
	72	Methyl benzoate	93-58-3	0.6	Cherry, strawberry, floral		0.74	
	73	Ethyl undecanoate	627-90-7	0.1	Orange, grapefruit, floral		1.34	
	74	Butyl 2-methylsalicylate	51115-63-0	0.3	Apple, peach, floral		0.65	
	75	Isobutyl decanoate	30673-38-2	0.2	Orange, lemon, floral		1.27	0.69
	76	Ethyl 8-nonenoate	5194-39-9	0.1	Orange, grapefruit, floral		44.23	
	77	Isoamyl decanoate	2306-91-4	0.2	Apple, banana, floral		5.61	3.11
	78	2-Methylbutyl decanoate	68067-33-4	0.07	Tropical fruit, oily		5.51	3.63
	79	Pentyl benzoate	2049-96-9	0.7	Cherry, strawberry, floral		40.12	13.86
	80	Resorcinol monobenzoate	136-36-7	30	Spicy, woody			
	81	Eugenyl acetate	93-28-7	0.01	Clove		15.88	
	82	Isoamyl laurate	6309-51-9	0.2	Apple, banana, floral		0.24	0.14
	83	2-Phenylethyl octanoate	5457-70-5	0.02	Rose		10.58	5.48
	84	Ethyl 9-hexadecenoate	54546-22-4	0.05	Apple, peach, floral			45.12
	85	Diethyl succinate	123-25-1	1	Apple, pear			2.32
	86	Monoethyl succinate	1070-34-4	0.5	Sweet, fruity, honey			4.21
	87	Ethyl caprylate	106-32-1	10	Citrus, floral			1.29
	88	Nonyl acetate	14936-66-4	0.2	Citrus, floral			0.52
	89	1-Methylbutyl acetate	626-38-0	0.3	Apple, strawberry, sweet			0.24
	90	Heptyl 2-methylbutanoate	50862-12-9	0.05	Tropical fruit, oily			0.56
	91	Ethyl 3-phenylpropanoate	2021-28-5	0.04	Sweet, honey, rose			1.31
	92	Isoamyl benzoate	94-46-2	0.7	Sweet, cherry, strawberry			20.43
	93	2-Phenylethyl formate	104-62-1	0.5	Sweet, honey, rose	0.61		0.23
Terpenes	1	Phellandrene	99-83-2	0.1	Citrus peel, green grass	16.87		
	2	(E)-3,7-dimethylocta-1,3,6-triene	3779-61-1	0.05	Rose, lavender, fruity	63.89		
	3	(2-Chloroethylsulfonylmethyl) benzene	66998-67-2	10	Onion, garlic, putrid	0.53		
	4	Ocimene	13877-91-3	5	Lemon, orange, floral, herbal	5.84		
	5	3-Carene	13466-78-9	20	Pine needle, resinous, fruity	1.57		
	6	2,6-Dimethyl-2,4,6-octatriene	673-84-7	0.03	Apple, strawberry, floral	43.59		
	7	Aristolene	6831-16-9	0.02	Spicy, woody	72.69		
	8	Styrene	100-42-5	1	Floral, plastic		9.22	
	9	Citronellol	106-22-9	0.5	Lemon, rose, herbal		0.38	
	10	Farnesol	4602-84-0	0.1	Floral, fruity, woody		1.03	
Phenols	1	2,4-Di-tert-butylphenol	96-76-4	0.5	Herbal, sweet	1.48	0.22	1.2
	2	(Z)-2-Methoxy-4-(1-propenyl) phenol/Isoeugenol	5912-86-7	0.05	Spicy, smoky, vanilla		3.19	1
	3	3,5-Di-tert-butylphenol	1138-52-9	0.2	Camphor, herbal, woody		0.45	
Ethers	1	Ethyl propyl ether	628-32-0	0.5	Ethereal, fruity	2.81		
	2	Isobutyl tert-butyl ether	33021-02-2	10	Fresh, sweet	2.26		
Aldehydes and ketones	1	3-Furaldehyde	498-60-2	20	Sweet, caramel, nutty	0.51		
	2	Heptanal	111-71-7	0.05	Fruity, green grass	80.37		
	3	Cyclohexanecarboxaldehyde	2043-61-0	0.1	Fruity, mint, camphor	1.1		
	4	Benzaldehyde	100-52-7	0.2	Sweet, bitter almond	33.65	5.72	
	5	Phenylacetaldehyde	122-78-1	2	Sweet, floral, honey	3.42	0.1	0.14
	6	trans-2-Nonenal	18829-56-6	0.02	Orange, lemon, oily	53.82		
	7	4-Ethylbenzaldehyde	4748-78-1	0.1	Sweet, cherry, strawberry, floral	14.57		
	8	3-Ethylbenzaldehyde	34246-54-3	0.1	Sweet, apple, peach, floral	9.89		
	9	3,4-dimethyl-benzaldehyde	5973-71-7	0.2	Cinnamon, clove, fruity	72.83	6.52	9.3
	10	Artemisia ketone	546-49-6	0.01	Herbal, camphor	19.29		
	11	1-Hepten-3-one	2918-13-0	0.05	Fruity, oily	2.17		
	12	6-Methyl-5-hepten-2-one	110-93-0	20	Apple, strawberry, floral	0.21		
	13	Heptanophenone	1671-75-6	0.1	Cherry, strawberry, cinnamon, woody	1.38		
	14	3-Hydroxyacetophenone	121-71-1	0.2	Sweet, herbal, honey, mint	0.11		
	15	Acetophenone	98-86-2	0.3	Sweet, almond, vanilla	2.50		
	16	4-Ethylacetophenone	937-30-4	0.2	Apple, peach, cinnamon	1.73		
	17	Damascenone	23726-93-4	0.1	Rose, violet, strawberry, raspberry	13.41		
	18	Geranylacetone	3796-70-1	0.04	Floral, lemon, rose, lavender	23.66	6.26	
	19	Benzophenone	119-61-9	0.1	Cherry, strawberry, cinnamon, woody	8.56		
	20	Isophthalaldehyde	626-19-7	0.1	Apple, peach, floral, woody		11.78	
	21	2H-Pyran-2,6(3H)-dione	5926-95-4	0.01	Sweet, caramel, fruity		10.37	43.16
	22	3-Methylpropiophenone	1772-30-6	1	Sweet, floral, honey, rose		0.1	
	23	2,3-Pentanedione	600-14-6	1	Creamy, buttery, nutty		4.94	
	24	2,4-Dimethylbenzaldehyde	15764-16-6	0.2	Strawberry, raspberry, floral			8.58
	25	Lauryl aldehyde	112-54-9	0.03	Fruity, waxy, fatty			4.2
	26	5-(Hydroxymethyl)dihydro-2(3H)-furanone	10374-51-3	0.05	Sweet, coconut, caramel			2.24

### 3.5. Evolution Characteristics of Flavor Profile of Composite Fruit Wine Before and After Fermentation

**Figure 6:**
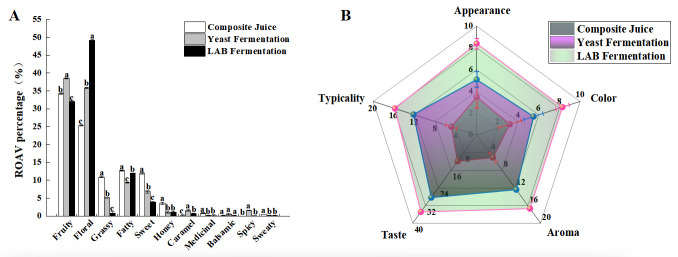
Flavor profile and sensory evaluation of sea buckthorn–grape blend wine. (**A**) Flavor profile; (**B**) sensory evaluation. Different letters above bars indicate statistically significant differences (*p* < 0.05) by one-way ANOVA followed by Duncan’s multiple range test.

According to the aroma classification method proposed by Ferreira et al. [28], the aromatic compounds were classified into 10 categories based on odor descriptors, to clarify the flavor characteristics of fruit wine at different stages. As shown in Figure 6A, the flavor of the sea buckthorn–grape composite juice was dominated by fruity aroma, while also presenting floral, grassy, fatty and sweet notes. After yeast fermentation, the flavor profile of the fruit wine was basically similar to that of the composite juice. After LAB fermentation, the flavor of the composite fruit wine was mainly concentrated on fruity, floral, fatty and sweet notes, among which floral aroma became the most prominent aroma type.

### 3.6. Sensory Evaluation Results of Composite Fruit Wine

The composite juice exhibited turbid appearance with poor transparency and obvious suspended solids (Figure 6B). It had a dull color, presenting dark red or light red, with weak aroma. The balance between fruity and wine aroma was poor, and the rancid off-flavor derived from sea buckthorn was relatively strong. The raw juice lacked sufficient fullness, with unbalanced sugar and acid, prominent bitterness, insufficient freshness, lack of unique flavor, and poor aftertaste freshness. Consequently, it received a low sensory score, with an average of 26.8 points.

After yeast alcoholic fermentation, the wine showed improved clarity with no obvious suspended solids, general gloss, and brighter color, presenting dark red or light red. The aroma was elegant, with good harmony between fruity and wine aroma. The rancid off-flavor derived from sea buckthorn was significantly reduced. The wine body was relatively full, with high acidity and mild bitterness. It still lacked sufficient flavor uniqueness, but had a refreshing aftertaste with good freshness. As a result, the sensory score increased significantly to an average of 63.6 points. After lactic acid bacteria fermentation, the wine was clear and transparent with gloss and no suspended solids, presenting bright color of dark red or light red. The aroma was elegant and pleasant, with harmonious fruity and wine aroma. The rancid off-flavor derived from sea buckthorn was extremely weak. The taste was mellow and full, with moderate sugar and acid, and light and refreshing mouthfeel. It featured unique flavor and fresh aftertaste, achieving the highest sensory score of 84.1 points. Notably, these sensory results were obtained from a trained panel of 10 assessors from a single institution, which may not fully represent the preferences of the broader consumer population, and thus the findings should not be overgeneralized to universal consumer acceptance.

## 4. Discussion and Conclusions

The dynamic changes in volatile aroma compounds during fermentation are the core factors determining the final quality of fruit wine. In this study, we observed significant differences in the volatile profile among the initial sea buckthorn–grape blend juice, yeast-fermented sample, and LAB-fermented sample. This significant shift may be attributed to the synergistic metabolic activities of yeast and LAB, as well as the enzymatic transformation of flavor precursors during the sequential fermentation process. In the initial blend juice, the detected volatile compounds were primarily free-form volatiles naturally derived from the raw sea buckthorn and grape fruits. However, a large portion of flavor precursors in the raw materials existed in a non-volatile bound state, which may not be detected via conventional headspace GC-MS analysis [2]. During the alcoholic fermentation stage, it is speculated that Saccharomyces cerevisiae can produce a series of new volatile metabolites through primary metabolism—including higher alcohols via the Ehrlich pathway from amino acid catabolism, and esters via alcohol acetyltransferase-mediated condensation reactions. Additionally, yeast may secrete hydrolytic enzymes such as β-glucosidase, which could catalyze the hydrolysis of partial bound flavor precursors and release free volatile compounds. These potential metabolic and enzymatic effects may account for the significant increase in both the number and total content of volatile compounds after yeast fermentation [27,29]. This phenomenon is consistent with previous findings in citrus wine and lychee wine fermentation, where yeast metabolism was confirmed to be the primary driver of the initial expansion of the volatile compound profile [30,31]. Similarly, Xu et al. [32] reported that synergistic co-fermentation of non-Saccharomyces yeasts significantly increased volatile aromatic compounds by 28.2% in citrus wine through upregulating glycolytic and fatty acid metabolic pathways, which further supports our observation that microbial metabolic synergy is the core mechanism for aroma enhancement during sequential fermentation.

Subsequently, during the fermentation stage conducted by LAB, the number of volatile compounds further increased despite a slight decrease in the total content of some volatile categories. This observation can be attributed to two key complementary processes: First, LAB strains are reported to possess hydrolytic enzymes including glycosidases and esterases, which may further hydrolyze yeast-untransformed bound flavor precursors and catalyze the cleavage of complex esters into smaller volatile molecules, potentially generating new detectable volatile compounds [33,34]. Second, LAB may perform catabolism of organic acids, amino acids, and residual sugars during fermentation, which could produce additional secondary metabolites to enrich volatile profile diversity [12,35]. These speculative mechanisms are consistent with our observed volatile compound variations but require further experimental verification. Similar observations have been reported in previous studies on sequential fermentation of fruit wines, where LAB fermentation was found to further increase the number of volatile compounds compared to yeast-only fermentation, primarily due to these complementary enzymatic and metabolic functions [30,33]. This progressive enrichment of volatile compounds across the fermentation stages directly contributed to the enhanced aroma richness of the final composite wine. The initial blend juice, with its limited volatile diversity, exhibited a relatively simple flavor profile dominated by raw fruit notes and off-flavors. In contrast, the sequential fermentation process gradually unlocked the flavor potential of the raw materials through microbial synergism, converting non-volatile precursors into diverse free volatiles and generating new metabolites, which ultimately resulted in a more complex and balanced aroma system.

Alcohols and esters are the core components that constitute the main aroma of composite fruit wine, and their evolution directly determines the aroma intensity and flavor hierarchy of fruit wine [29,36]. Combined with the analysis of Figure 2 and Table S1, in the yeast fermentation stage, both the number and relative content of alcohols increased significantly, among which 1-pentanol, isoamyl alcohol, (2S,3S)-(+)-2,3-butanediol, 2-(methylthio)ethanol and 3-(methylthio)-1-propanol were present at high levels. These compounds were primarily derived from the biotransformation of organic acids and amino acids by yeast, which is consistent with previous findings on aroma evolution during high-acid fruit wine fermentation [37]. In the LAB fermentation stage, the relative content of alcohols decreased while the compound number further increased. Based on previous fermentation mechanism studies, it is hypothesized that this phenomenon results from bacterial metabolic transformation: partial alcohols may be consumed via esterification and aldehyde–ketone reactions, and ester decomposition may promote the generation of new alcohols. This trend is consistent with previous research conclusions on microbial-driven ester metabolism during fermentation [34]. As an important component of volatile aroma in fruit wine, esters are formed by the condensation of hydroxyl groups of alcohols and phenols and carboxyl groups of organic acids, which have a direct impact on sensory quality [38,39]. Combined with the analysis of Figure 3 and Table S1, in the yeast fermentation stage, the relative content and number of esters showed an upward trend, which effectively enhanced the aroma intensity and flavor hierarchy of fruit wine. A similar phenomenon was also observed in a previous fermentation study of “Zaoheibao” grape wine [38]. In the LAB fermentation stage, the relative content of esters decreased, but their number was further enriched, which enhanced the aroma richness of the fruit wine.

Although terpenes and phenol ethers account for a relatively low proportion in the system, they play an important auxiliary role in coordinating and strengthening the overall flavor of fruit wine. Terpenes mainly include monoterpenes and sesquiterpenes, among which monoterpenes can endow fruit wine with typical floral and fruity aromas [40]. Combined with the analysis of Figure 5 and Table S1, the relative content of terpenes decreased significantly during the yeast fermentation stage, followed by a slight rebound as fermentation progressed. This trend aligns with the previously reported aroma evolution pattern during lychee wine fermentation [31]. Additionally, the type composition of terpenes changed significantly after fermentation compared with that before fermentation. In the LAB fermentation stage, the relative content of terpenes further decreased, but there was no significant difference in the number of terpenes compared with the yeast fermentation stage, leading to a weak overall impact on the final flavor. Phenols and ethers, on the other hand, can coordinate and strengthen the overall aroma of fruit wine [41]. Combined with the analysis of Figure 6 and Table S1, during the yeast fermentation process, the relative content of phenols and ethers gradually decreased while the compound number increased, with a significantly altered composition relative to the initial juice. During the LAB fermentation stage, the relative content of phenols and ethers rebounded, accompanied by distinct compositional differences from the initial juice and yeast-fermented samples. It is speculated that the increased content and diversity of phenols and ethers may help strengthen and coordinate the overall aroma characteristics of composite fruit wine.

From the perspective of the evolution of ROAV and flavor types, the co-fermentation process realized the targeted optimization of the system’s flavor. In the initial composite juice, off-flavor acid substances such as valeric acid and nonanoic acid, alongside certain high-abundance terpenes, aldehydes, and ketones, exhibited high ROAVs. These compounds were the core drivers of the initial system’s poor odor. After yeast fermentation, the ROAVs of these off-flavor substances decreased significantly, while the contribution of key aroma substances such as 1-pentanol and 3-methylbutyl 3-methylbutanoate rose substantially, initially completing the flavor improvement. After entering the LAB fermentation stage, the ROAVs of off-flavor substances further decreased, and floral and fruity substances such as trans-nerolidol, ethyl decanoate and ethyl laurate became the main contributing components of the flavor. Finally, the flavor characteristics of the composite fruit wine changed from the initial fruity-dominated profile to a floral-core profile, while retaining coordinated fatty and sweet notes, realizing the flavor upgrading.

This study quantified volatile compounds using relative abundance via peak area normalization without absolute calibration. While the relative ratios and dynamic trends across fermentation stages are reliable due to consistent analytical conditions, the lack of absolute quantification precludes direct comparison with studies using absolute methods. Additionally, variations in mass spectrometry response factors may affect the accuracy of relative content and aroma contribution assessments. Furthermore, this study did not perform direct microbiological population dynamics analysis or targeted metabolic flux analysis, which limits our ability to precisely elucidate the specific microbial metabolic pathways driving the observed aroma changes. Nevertheless, these limitations do not invalidate the core findings of this study. Specifically, the peak area normalization approach reliably supports relative comparisons of volatile compound abundances between fermentation stages and the interpretation of temporal evolution patterns of the overall aroma profile, as all samples were processed and analyzed under identical experimental conditions. However, we acknowledge that aroma contribution estimates based on ROAV should be interpreted with appropriate caution, as they are derived from relative rather than absolute concentrations and may be affected by variations in mass spectrometry response factors.

In summary, by systematically analyzing the dynamic changes of volatile aroma components during the fermentation of sea buckthorn–grape composite fruit wine, this study clarified the improvement effects of the co-fermentation of yeast and LAB on the off-flavor of the initial raw materials, as well as its construction effect on the aroma system of fruit wine. During the co-fermentation process, off-flavor substances (acids, aldehydes, and ketones) were gradually reduced, and the content and species of key aroma-active alcohols and esters were substantially enriched. Terpenes and phenol ethers also exhibited dynamic variations throughout fermentation. These dynamic compound changes may collectively contribute to the flavor optimization of composite fruit wine, and the synergistic regulatory effects of sequential fermentation are hypothesized to be the core driving factor for flavor improvement. The results of this study can provide a reliable theoretical basis for the flavor regulation and new product development of sea buckthorn products, help to improve the utilization rate of sea buckthorn raw materials, and promote the development of related industries.

## Figures and Tables

**Table 1:** Sensory evaluation criteria of sea buckthorn–grape composite fruit wine at different fermentation stages.

Item	Scoring Criteria	Score
Appearance (10 points)	Clear, transparent and glossy, without suspended solids	8~10
	Good clarity without obvious suspended solids, moderate gloss	4~7
	Poor clarity and gloss with obvious suspended solids	0~4
Color (10 points)	Bright color, dark red or light red	8~10
	Relatively bright color, dark red or light red	4~7
	Dull color, dark red or light red	0~4
Aroma (20 points)	Elegant and pleasant aroma with harmonious fruit and wine notes; extremely weak rancid off-flavor of sea buckthorn	14~20
	Elegant aroma with good harmony of fruit and wine notes; slight rancid off-flavor of sea buckthorn	7~13
	Inferior aroma with poor balance of fruit and wine notes; heavy rancid off-flavor of sea buckthorn	0~6
Taste (40 points)	Mellow and full wine body, moderate sour-sweet taste, fresh and crisp mouthfeel	32~40
	Relatively full wine body, high acidity and slight bitterness	19~31
	Insufficient fullness of wine body, unbalanced sour and sweet taste, strong bitterness and poor refreshing sensation	0~18
Typicality (20 points)	Unique flavor and fresh aftertaste	16~20
	Less distinctive flavor, fresh and pleasant aftertaste	9~15
	Lack of unique flavor and unsatisfactory fresh aftertaste	0~8

## Data Availability

The original contributions presented in this study are included in the article/Supplementary Materials. Further inquiries can be directed to the corresponding author.

## Supplementary Materials

The following supporting information can be downloaded at: https://www.mdpi.com/article/10.3390/foods15132297/s1, Table S1: Aromatic compounds in fruit wines produced by fermenting blended juices with different bacterial strains.

## Abbreviations

The following abbreviations are used in this manuscript:

GC	Gas Chromatography
HS-SPME-GC-MS	Headspace Solid Phase Microextraction-Gas Chromatography-Mass Spectrometry
LAB	lactic acid bacteria
MS	Mass Spectrometry
OPLS-DA	orthogonal partial least squares discriminant analysis
SD	standard deviation
ROAV	relative odor activity value
