# Supplementary material for: Unraveling the Dynamic Evolution of Volatile Aroma Compounds in Sea Buckthorn–Grape Composite Fruit Wine During Sequential Yeast–Lactic Acid Bacteria Fermentation
Source: Foods. 2026 Jun 26;15(13):2297. doi: 10.3390/foods15132297 (PMC13361229; doi:10.3390/foods15132297)
Supplement: Supplementary file 1 [file foods-15-02297-s001.zip › foods-4382162-supplementary.pdf]

Supplementary materials

Table S1. Aromatic compounds in fruit wines produced by fermenting blended juices with different bacterial strains

Name	CAS Number	Relative content (%)		
		Composite juice	Yeast fermentation	LAB fermentation
(Methylthio)acetic acid	2444-37-3	0.11±0.02[a]	-	0.09±0.05[b]
Isovaleric acid	503-74-2	0.5±0.10[a]	-	-
2-Hydroxy-3-methylbutanoic acid	4026-18-0	0.08±0.02	-	-
Valeric acid	109-52-4	1.8±0.40[a]	0.48±0.26[b]	0.43±0.22[c]
2-Acetoxy-3-methylbutanoic acid	18667-97-5	0.07±0.02	-	-
Heptanoic acid	111-14-8	0.29±0.01	-	-
Octanoic aci	124-07-2	1.59±0.04[b]	1.85±0.11[a]	0.22±0.14[c]
Nonanoic acid	112-05-0	1.73±0.23[a]	0.06±0.05[c]	0.14±0.00[b]
cis-5-Dodecenoic acid	2430-94-6	3.90±0.17[a]	1.81±0.16[b]	1.77±0.07[c]
Hexanoic acid	142-62-1	-	0.56±0.00[b]	0.59±0.01[a]
2-Methylhexanoic acid	4536-23-6	-	0.07±0.03	-
Decanoic acid	334-48-5	-	3.15±0.22[a]	1.13±0.09[b]
Lauric acid	143-07-7	-	0.20±0.14	-
2-Methylhexanoic acid	116-53-0	-	-	0.20±0.04
Phenylacetic acid	103-82-2	-	-	0.01±0.00
Ethyl (Z)-4-decenoate	7367-84-2	-	-	0.34±0.05
3,4-Dimethylpentanoic acid	3302-06-5	-	0.12±0.09	-
1-Octanol	111-87-5	0.33±0.03	-	-
Phenylethyl alcohol	60-12-8	0.67±0.17	-	-
4-Penten-2-ol	625-31-0	-	0.05±0.02	-
1-Pentanol	71-41-0	-	15.92±0.34	-
2-Methylbutanol	137-32-6	-	0.54±0.51	-
Isoamyl alcohol	123-51-3	-	0.97±0.11	-
(2S,3S)-(+)-2,3-Butanediol	19132-06-0	-	0.24±0.18[a]	0.1±0.01[b]
2-(Methylthio)ethanol	5271-38-5	-	0.19±0.02	-
3-(Methylthio)propanol	505-10-2	-	0.13±0.11[a]	0.13±0.00[a]
1-Methoxy-2-methyl-2-propanol	3587-64-2	-	-	0.04±0.01
4-Methyl-2-heptanol	56298-90-9	-	-	0.19±0.03
Benzyl alcohol	100-51-6	-	-	0.05±0.01
Isophytol	505-32-8	-	0.02±0.00	
1-Heptanol	111-70-6	-	-	0.08±0.07
(S)-(+)-6-Methyl-1-octanol	110453-78-6	-	-	0.17±0.01
5-Methyl-2,3-dihydro-1H-inden-4-ol	20294-31-9		-	0.07±0.06
6-Methylindan-4-ol	20294-32-0	--	-	0.08±0.05
1,14-Tetradecanediol	19812-64-7	-	-	0.03±0.03
1-Adamantanol	768-95-6	-	-	0.01±0.00
R)-(+)-β-Citronellol	1117-61-9	-	-	0.09±0.02
trans-Nerolidol	40716-66-3	-	-	0.08±0.04
Methyl propionate	554-12-1	1.27±0.05	-	-
Ethyl isovalerate	108-64-5	3.56±0.51		-

Table S1. Aromatic compounds in fruit wines produced by fermenting blended juices with different bacterial strains (continued)

Name	CAS Number	Relative content (%)		
		Composite juice	Yeast fermentation	LAB fermentation
Methyl acetoacetate	13865-19-5	1.94±0.17	-	-
Propyl isovalerate	557-00-6	0.24±0.17	-	-
Ethyl hexanoate	123-66-0	13.03±0.06[a]	5.37±0.04[b]	3.72±0.14[c]
Vinyl acrylate	2177-18-6	-	-	0.15±0.04[c]
Isobutyl isovalerate	589-59-3	0.42±0.27	-	-
Isoamyl isobutyrate	2050-01-3	0.92±0.17[a]	0.05±0.01[b]	-
Isoamyl butyrate	106-27-4	1.07±0.08[a]	0.08±0.01[b]	-
2-Methylbutyl butyrate	51115-64-1	0.80±0.00	-	-
cis-3-Hexenyl isovalerate	35154-45-1	0.40±0.01	-	-
3-Methylbutyl 2-methylbutanoate	27625-35-0	1.94±0.07	0.31±0.24	-
Isoamyl isovalerate	659-70-1	1.83±0.04[c]	9.37±0.17[a]	3.21±0.11[b]
Methyl octanoate	111-11-5	0.66±0.55	-	-
Butyl hexanoate	626-82-4	0.36±0.27	-	-
Pentyl 3-methylbutanoate	25415-62-7	0.39±0.34[a]	0.04±0.0.1[a]	-
Ethyl benzoate	93-89-0	7.22±0.28[a]	0.77±0.16[c]	0.88±0.17[b]
Hexyl 2-methylbutanoate	10032-13-0	1.60±0.11[a]	0.17±0.1[b]	0.07±0.01[c]
Furfuryl valerate	36701-01-6	0.64±0.32[a]	-	0.04±0.02[b]
Furfuryl isovalerate	13678-60-9	0.70±0.04[a]	0.08±0.08[b]	0.06±0.03[c]
Ethyl phenylacetate	101-97-3	2.85±0.28[a]	0.31±0.14[b]	0.15±0.06[c]
2-Methylbutyl hexanoate	2601-13-0	4.95±0.04[a]	0.96±0.17[b]	0.21±0.11[c]
Propyl benzoate	2315-68-6	0.20±0.19[a]	0.03±0.02[c]	0.64±0.17[b]
Pentyl hexanoate	540-07-8	0.15±0.06[a]	0.02±0.00[b]	-
Propyl octanoate	624-13-5	0.12±0.06[a]	0.06±0.03[b]	-
Ethyl nonanoate	123-29-5	0.29±0.05[c]	0.76±0.17[a]	0.64±0.21[b]
Isobutyl benzoate	120-50-3	0.06±0.01[b]	0.01±0.00[c]	0.07±0.05[a]
2-Hydroxyethyl benzoate	94-33-7	0.06±0.03[a]	-	0.02±0.00[b]
Isoamyl 2-furoate	615-12-3	0.17±0.05	-	-
Methyl decanoate	110-42-9	0.10±0.01[a]	0.03±0.01[b]	-
Heptyl isovalerate	56423-43-9	0.33±0.20[a]	0.06±0.05[b]	-
cis-3,7-Dimethyl-2,6-octadien-1-yl acetate	141-12-8	0.16±0.04	-	-
Butyl benzoate	136-60-7	0.03±0.01[b]		0.08±0.01[a]
Ethyl trans-4-decenoate	76649-16-6	3.35±0.11[a]	1.13±0.24[b]	0.36±0.26[c]
Hexyl hexanoate	6378-65-0	0.19±0.18	-	-
Citronellyl formate	105-85-1	-	0.02±0.00[b]	0.1±0.10[a]
Ethyl decanoate	110-38-3	4.40±0.61[b]	0.88±0.12[c]	14.82±0.17[a]
Benzyl valerate	10361-39-4	2.89±0.21[a]	0.73±0.02[b]	-
Isoamyl octanoate	2035-99-6	2.86±0.05[a]	1.82±0.09[b]	1.36±0.07[c]
2-Methylbutyl octanoate	67121-39-5	0.62±0.08[a]	0.60±0.1[ab]	0.4±0.02[c]
Ethyl 2,4-decadienoate	3025-30-7	0.15±0.10[a]	0.09±0.04[b]	0.05±0.00[c]
2-Phenylethyl 2-methylbutanoate	24817-51-4	2.81±0.13[a]	1.30±0.28[b]	0.84±0.14[c]
Pentyl salicylate	2050-08-0	0.20±0.08[c]	0.07±0.02[a]	0.03±0.01[b]

Table S1. Aromatic compounds in fruit wines produced by fermenting blended juices with different bacterial strains (continued)

Name	CAS Number	Relative content (%)		
		Composite juice	Yeast fermentation	LAB fermentation
Ethyl laurate	106-33-2	0.89±0.55[c]	6.05±0.61[b]	9.87±0.17[a]
2-Phenylethyl hexanoate	6290-37-5	0.86±0.19[b]	1.06±0.33[a]	-
Benzyl benzoate	120-51-4	0.06±0.01[a]	0.03±0.01[b]	0.01±0.00[c]
Ethyl myristate	124-06-1	0.10±0.10[c]	0.41±0.21[b]	1.04±0.15[a]
2-Phenylethyl benzoate	94-47-3	0.04±0.00[b]	-	0.08±0.04[a]
ethyl cis-9-hexadecenoate	56219-10-4	0.28±0.11[c]	0.26±0.17[b]	1.71±0.16[a]
Ethyl palmitate	628-97-7	0.41±0.27[b]	0.21±0.14[c]	1.23±0.32[a]
Propyl lactate	616-09-1	-	2.75±0.01[a]	0.04±0.01[b]
Methyl isothiocyanate	556-61-6	-	0.04±0.00	-
Methyl formate	107-31-3	-	0.05±0.01[b]	1.42±0.26[a]
Isoamyl acetate	123-92-2	-	6.62±0.07[a]	4.26±0.29[b]
Resorcinol monobenzoate	136-36-7	-	0.05±0.04[a]	0.01±0.00[b]
Methyl dithioacetate	2168-84-5	-	0.08±0.01	-
Hexyl acetate	142-92-7	-	0.06±0.04	-
Ethyl 2-furoate	614-99-3	-	0.17±0.02[a]	0.11±0.07[b]
Methyl hydroxypivalate	14002-80-3	-	0.08±0.26	-
Ethyl heptanoate	106-30-9	-	0.20±0.11[ab]	0.26±0.08[a]
Isoamyl valerate	2050-09-1	-	0.19±0.14[a]	0.1±0.02[b]
2-Methylbutyl isovalerate	2445-77-4	-	0.02±0.00	-
Isobutyl hexanoate	105-79-3	-	0.07±0.01	-
Octyl 2-methylbutanoate	29811-50-5	-	0.08±0.020	-
Hexyl acetoacetate	13562-84-0	-	0.14±0.05	-
Isoamyl caproate	2198-61-0	-	4.62±0.19[a]	1.43±0.24[b]
Ethyl myristate	103-45-7	-	3.49±0.16[b]	6.74±0.14[a]
Heptyl acetate	112-06-1	-	0.05±0.04	-
Nonyl acetate	143-13-5	-	0.07±0.07	-
Isopentyl octanoate	2035-99-6	-	0.25±0.11[a]	0.07±0.05[b]
Pentyl octanoate	638-25-5	-	0.28±0.26[a]	0.11±0.04[b]
Geranyl isovalerate	109-20-6	-	0.03±0.01	-
Ethyl dec-9-enoate	67233-91-4	-	1.14±0.00[a]	0.95±0.26[b]
Methyl 2-oxobutanoate	3952-66-7	-	0.30±0.21	-
2-Phenylethyl isovalerate	140-26-1	-	0.11±0.11[b]	0.8±0.32[a]
Methyl benzoate	93-58-3	-	0.16±0.15	-
Ethyl undecanoate	627-90-7	-	0.05±0.02	-
Butyl 2-methylsalicylate	51115-63-0	-	0.07±0.01	-
Isobutyl decanoate	30673-38-2	-	0.09±0.04[a]	0.09±0.02[a]
2,2,4-Trimethyl-1,3-pentanediol diisobutyrate	6846-50-0	-	-	0.21±0.14
Ethyl 8-nonenoate	5194-39-9	-	1.55±0.24	-
Isoamyl decanoate	2306-91-4	-	0.39±0.05[b]	0.42±0.09[a]
2-Methylbutyl decanoate	68067-33-4	-	0.13±0.03[b]	0.17±0.02[a]
Pentyl benzoate	2049-96-9	-	9.81±0.08[a]	6.54±0.21[b]
Eugenyl acetate	93-28-7	-	0.06±0.00	

Table S1. Aromatic compounds in fruit wines produced by fermenting blended juices with different bacterial strains (continued)

Name	CAS Number	Relative content (%)		
		Composite juice	Yeast fermentation	LAB fermentation
Isoamyl laurate	6309-51-9	-	0.02±0.00^{a}	0.02±0.00^{a}
2-Phenylethyl octanoate	5457-70-5	-	0.07±0.01^{a}	0.07±0.01^{a}
Ethyl 9-hexadecenoate	54546-22-4	-	-	1.52±0.03
Ethyl acetate	141-78-6	-	-	0.1±0.07
Methyl lactate	547-64-8	-	-	0.26±0.14
Vinyl cinnamate	17719-70-9	-	-	0.1±0.05
Dimethyl carbonate	616-38-6	-	-	0.01±0.00
Phenacyl formate	55153-12-3	-	-	0.03±0.01
1-Hydroxypropan-2-yl acetate	6214-01-3	-	-	0.07±0.01
Vinyl sorbate	42739-26-4	-	-	0.04±0.02
Diethyl succinate	123-25-1	-	-	1.56±0.14
Monoethyl succinate	1070-34-4	-	-	1.42±0.26
Ethyl caprylate	106-32-1	-	-	8.66±0.11
2-ethylhexyl pivalate	16387-18-1	-	-	0.12±0.03
Methyl 3-hydroxytetradecanoate	55682-83-2	-	-	0.13±0.03
Diisopentyl carbonate	2050-95-5	-	-	0.02±0.00
Nonyl acetate	14936-66-4	-	-	0.07±0.01
1-Methylbutyl acetate	626-38-0	-	-	0.05±0.04
Heptyl 2-methylbutanoate	50862-12-9	-	-	0.02±0.01
Ethyl 3-phenylpropanoate	2021-28-5	-	-	0.04±0.01
Isoamyl benzoate	94-46-2	-	-	9.64±0.17
2-Phenylethyl formate	104-62-1	-	-	0.02±0.01
Methyl 10-methylundecanoate	5129-56-6	-	-	0.02±0.00
Butylparaben	94-26-8	-	-	0.01±0.00
Ethyl ricinoleate	55066-53-0	-	-	0.02±0.00
Ethyl elaidate	6114-18-7	-	-	0.02±0.01
4,4-Dimethyl-1-pentene	762-62-9	0.08±0.03		
Phellandrene	99-83-2	0.22±0.05		
(E)-3,7-dimethylocta-1,3,6-triene	3779-61-1	0.42±0.17		
(2-Chloroethylsulfonylmethyl)benzene	66998-67-2	0.69±0.09		
Ocimene	13877-91-3	3.81±0.11		
3-Carene	13466-78-9	4.10±0.26^{a}	0.48±0.22^{b}	-
2,6-Dimethyl-2,4,6-octatriene	673-84-7	0.17±0.02	-	-
7-Tetradecene	10374-74-0	0.11±0.07	-	-
Aristolene	6831-16-9	0.19±0.08	-	-
Styrene	100-42-5	-	3.22±0.26	-
3-Ethyl-2-methyl-1-heptene	19780-60-0	-	-	0.03±0.02
Citronellol	106-22-9	-	0.07±0.05^{a}	0.05±0.02^{b}
Farnesol	4602-84-0	-	0.04±0.01^{b}	0.05±0.01^{a}
α-Calacorene	21391-99-1	-	-	0.02±0.01
3-Furaldehyde	498-60-2	1.32±0.19	-	-
Heptanal	111-71-7	0.52±0.17	-	-

Cyclohexanecarboxaldehyde	2043-61-0	0.01±0.00	-	-

Table S1. Aromatic compounds in fruit wines produced by fermenting blended juices with different bacterial strains (continued)

Name	CAS Number	Relative content (%)		
		Composite juice	Yeast fermentation	LAB fermentation
Benzaldehyde	100-52-7	0.88±0.22^{a}	0.4±0.35^{b}	-
Phenylacetaldehyde	122-78-1	0.89±0.19^{a}	0.07±0.05^{c}	0.19±0.07^{b}
trans-2-Nonenal	18829-56-6	0.14±0.02	-	-
4-Ethylbenzaldehyde	4748-78-1	0.19±0.14	-	-
3-Ethylbenzaldehyde	34246-54-3	0.13±0.06	-	-
3,4-dimethyl-benzaldehyde	5973-71-7	1.90±0.28^{a}	0.46±0.11^{c}	1.25±0.28^{b}
Isophthalaldehyde	626-19-7	-	0.41±0.28^{a}	0.01±0.00^{b}
2,4-Dimethylbenzaldehyde	15764-16-6	-	-	1.16±0.15
Lauryl aldehyde	112-54-9	-	-	0.08±0.01
Pentadecanal	2765/11/9	-	-	0.08±0.03
Artemisia ketone	546-49-6	0.03±0.01	-	-
1-Hepten-3-one	2918-13-0	0.01±0.00	-	-
6-Methyl-5-hepten-2-one	110-93-0	0.55±0.37	-	-
2H-Pyran-2,6(3H)-dione	5926-95-4	-	0.04±0.01^{b}	0.29±0.09^{a}
Heptanophenone	1671-75-6	0.02±0.00	-	-
3-Hydroxyacetophenone	121-71-1	-	-	0.01±0.00
Acetophenone	98-86-2	0.10±0.08	-	-
4-Ethylacetophenone	937-30-4	0.05±0.03	-	-
Damascenone	23726-93-4	0.17±0.09	-	-
Geranylacetone	3796-70-1	0.12±0.05^{a}	0.09±0.03^{b}	-
Benzophenone	119-61-9	0.11±0.01	-	-
3-Methylpropiophenone	1772-30-6	-	0.04±0.10	-
2,3-Pentanedione	600-14-6	-	1.73±0.26^{a}	0.02±0.00^{b}
Acetone-d6	666-52-4	-	-	0.02±0.01
1-(2,4-dihydroxy-3-methylphenyl)propan-1-one	63876-46-0	-	-	0.01±0.00
5-(Hydroxymethyl)dihydro-2(3H)-furanone	10374-51-3	-	-	0.08±0.06
2,4-Dimethylpropiophenone	50390-51-7	-	-	0.06±0.10
Eugenol	97-53-0	0.22±0.07	-	-
(Z)-2-Methoxy-4-(1-propenyl) phenol / Isoeugenol	5912-86-7	-	0.06±0.01^{a}	0.03±0.25^{b}
2,4-Di-tert-butylphenol	96-76-4	0.1±0.01^{b}	0.04±0.00^{c}	0.41±0.14^{a}
3,5-Di-tert-butylphenol	1138-52-9	-	0.03±0.01	-
Isobutyl tert-butyl ether	33021-02-2	2.94±0.09	-	-
Ethyl propyl ether	628-32-0	0.18±0.03	-	-
2-Chloroethyl methyl ether	627-42-9	-	-	0.03±0.00
Allyl butyl ether	3739-64-8	-	-	0.03±0.01
tert-Butyl glycidyl ether	7665-72-7	-	-	0.38±0.14
tert-Amyl methyl ether	994-05-8	-	-	0.05±0.01
